# NADPH Oxidase 2-Mediated Insult in the Auditory Cortex of Zucker Diabetic Fatty Rats

**DOI:** 10.1155/2019/3591605

**Published:** 2019-07-30

**Authors:** Zheng-De Du, Wei Wei, Shukui Yu, Qing-Ling Song, Ke Liu, Shu-Sheng Gong

**Affiliations:** ^1^Department of Otorhinolaryngology, Beijing Friendship Hospital, Capital Medical University, 95 Yongan Road, Xicheng District, Beijing 100050, China; ^2^Department of Otology, Shengjing Hospital, China Medical University, 36 Sanhao Street, Heping District, Shenyang 110004, China; ^3^Department of Otorhinolaryngology, 731 Hospital, China Aerospace Science and Industry Company, No. 3 Courtyard, Gangnanli, Yungang Town, Fengtai District, Beijing 100074, China

## Abstract

Clinical data has confirmed that auditory impairment may be a secondary symptom of type 2 diabetes mellitus (T2DM). However, mechanisms underlying pathologic changes that occur in the auditory system, especially in the central auditory system (CAS), remain poorly understood. In this study, Zucker diabetic fatty (ZDF) rats were used as a T2DM rat model to observe ultrastructural alterations in the auditory cortex and investigate possible mechanisms underlying CAS damage in T2DM. The auditory brainstem response (ABR) of ZDF rats was found to be markedly elevated in low (8 kHz) and high (32 kHz) frequencies. Protein expression of NADPH oxidase 2 (NOX2) and its matching subunits P22^phox^, P47^phox^, and P67^phox^ was increased in the auditory cortex of ZDF rats. Expression of 8-hydroxy-2-deoxyguanosine (8-OHdG), a marker of DNA oxidative damage, was also increased in the neuronal mitochondria of the auditory cortex of ZDF rats. Additionally, decreases in the mitochondrial total antioxidant capabilities (T-AOC), adenosine triphosphate (ATP) production, and mitochondrial membrane potential (MMP) were detected in the auditory cortex of ZDF rats, suggesting mitochondrial dysfunction. Transmission electron microscopy results indicated that ultrastructural damage had occurred to neurovascular units and mitochondria in the auditory cortex of ZDF rats. Furthermore, cytochrome c (Cyt c) translocation from mitochondria to cytoplasm and caspase 3-dependent apoptosis were also detected in the auditory cortex of ZDF rats. Consequently, the study demonstrated that T2DM may cause morphological damage to the CAS and that NOX2-associated mitochondrial oxidative damage and apoptosis may be partly responsible for this insult.

## 1. Introduction

Diabetes mellitus is a significant health risk to neural and microvascular diseases [1]. However, there have been some controversial studies regarding the effect of diabetes on hearing [2]. During the last decade, epidemiological and scientific evidences have indicated a close relationship between sensorineural hearing loss and type 2 diabetes mellitus (T2DM) [3], where T2DM is associated with bilateral sensorineural hearing loss, especially at high frequencies [4]. However, detailed pathophysiology associated with hearing loss resulting from diabetes is still unclear [5]. The validity of studies suggesting that microangiopathy in the inner ear [6], neuronal degeneration [7], diabetic encephalopathy, and oxygen-free radical hyperactivity [6, 8] may play important roles in the process of hearing loss induced by diabetes is still being debated.

Oxidative stress might play an important role in the impairment of hearing in T2DM patients [8]. In our previous study [9], we fed rats a high-fat diet for 12 months and found that the generation of reactive oxygen species (ROS) increased in the cochlea and the main source of ROS in the cochlea was from the NADPH oxidase (NOX) system. Furthermore, we demonstrated that NOX-induced mitochondrial oxidative damage in the cochlea might be partly responsible for the decline of auditory function in the rats. Apart from mitochondria, the NOX system is another essential ROS-generating site, which contains NOX1, NOX2, NOX3, NOX4, NOX5, DUOX1, and DUOX2 [10, 11]. The NOX system transports electrons across the plasma membrane and generates superoxide to produce downstream ROS [12]. In the NOX system, NADPH oxidase 2 (NOX2) is the most important NOX and widely locates in phagocytic cells and neurons [10, 11]. NOX2 matches with subunits P22^phox^, P47^phox^, and P67^phox^ to form the active NOX2 enzyme complex, which transports electrons from cytoplasmic NADPH to phagosomal or extracellular oxygen to generate ROS, such as superoxide anions, hydroxyl radicals, and hydrogen peroxide [13]. Among these subunits, P47^phox^ plays a vital role in the functioning of the NOX2 enzyme complex [14]. However, the purpose behind the expression of NOX2 and its matching subunits in the central auditory system (CAS) of T2DM remains unclear.

The Zucker diabetic fatty (ZDF) rat is an inbred T2DM rat model that closely mimics human adult-onset diabetes (type 2) and related complications. Recently, ZDF rats have been used to investigate the effects of T2DM on different cells and tissues [15–19], including the cochlea [20]. In this study, the ZDF rats were used to investigate CAS impairment in T2DM. We analyzed auditory function, protein expression of NOX2 and its matching subunits P22^phox^, P47^phox^, and P67^phox^, mitochondrial and cytoplasmic cytochrome c (Cyt c), protein levels of cleaved caspase 3 (C-cas3), DNA oxidative damage marker 8-hydroxy-2-deoxyguanosine (8-OHdG) [21], ultrastructural alteration, levels of mitochondrial total antioxidant capabilities (T-AOC), adenosine triphosphate (ATP) production, mitochondrial membrane potential (MMP), and the apoptotic cells in the auditory cortex of ZDF rats. Furthermore, we searched for a possible mechanism underlying the impairment of CAS by T2DM.

## 2. Experimental Procedures

### 2.1. Animals

The homozygous (fa/fa) ZDF rat is an inbred T2DM rat model. Homozygous dominant (+/+) and heterozygous (fa/+) lean genotypes remain normoglycemic. The rats were divided into two groups (*n* = 12 per group): the ZDF group consisting of homozygous dominant rats (fa/fa) and the control group consisting of heterozygous dominant rats (fa/+). Both groups received similar nutritive supplementation until the age of 36 weeks. All procedures followed the Guideline for Care and Use of Laboratory Animals of the National Institutes of Health. The experimental protocol was approved by the Committee on the Ethics of Animal Experiments of the Capital Medical University.

### 2.2. Auditory Brainstem Response (ABR)

ABR detected auditory function of 36-week-old rats in the ZDF (*n* = 6) and control (*n* = 6) groups. Rats were anaesthetized with ketamine (30 mg/kg, intraperitoneal injection) and chlorpromazine (15 mg/kg, intramuscular injection). The sound delivery tube of an insert earphone was tightly fitted into the external auditory canal. The active lead electrode needle was subcutaneously positioned at the vertex, and the reference electrode was positioned on the top of the nose. The ground electrodes were positioned over the neck muscles. The ABR threshold was recorded in an electrically shielded, double-walled, and radio frequency-shielded sound booth. The response was measured using a tone burst stimulus at 8, 16, and 32 kHz with a computer-based signal averaging system (Tucker-Davis Technologies, USA). The decibel level was raised in 5 dB steps from 20 dB below the threshold up to 90 dB sound pressure level (SPL). Following visual inspection of stacked waveforms, the lowest SPL intensity capable of eliciting a replicable, visually detectable response that displayed at least two peaks and a minimum amplitude of 0.5 *μ*V, the lowest SPL at any wave that could be detected was considered as the ABR threshold. Thresholds were verified by two unblinded investigators.

### 2.3. Western Blotting

Following the acoustic testing, four rats in each group were sacrificed and both hemispheres of the auditory cortex of each rat were removed. Mitochondrial and cytosolic fractions were separated with a commercially available cytosol/mitochondria fractionation kit (Beyotime, China), and protein was extracted using a RIPA lysis buffer (Beyotime, China) according to the manufacturer's protocol. An Enhanced BCA Protein Assay Kit (Beyotime, China) was used to measure protein concentrations in the supernatant of the homogenate. Following protein measurement, 25 *μ*g of each protein lysate was separated using 12% SDS-polyacrylamide gels and transferred to polyvinylidene difluoride (PVDF) membranes. The membranes were blocked in 5% (*w*/*v*) nonfat milk in Tris-buffered saline (TBS) for 1 h, washed briefly in TBS, and incubated overnight at 4°C with appropriately diluted primary antibodies as follows: anti-NOX2 (1 : 200; Servicebio, China), anti-P22^phox^ (1 : 500; Abcam, USA), anti-P47^phox^ (1 : 500; Abcam, USA), anti-P67^phox^ (1 : 500; Abcam, USA), anti-Cyt c (1 : 500; Abcam, USA), anti-C-cas3 (1 : 1000; Cell Signaling Technology, USA), anti-*β*-actin antibody (1 : 1000; Servicebio, China), or anti-cytochrome c oxidase (COX) IV antibody (1 : 1000; Proteintech, China). The membranes were washed to remove excess primary antibodies before being incubated for 1 h at room temperature with appropriate horseradish peroxidase- (HRP-) conjugated secondary antibody (1 : 5000; ZSGB-BIO, China). Membranes were visualized using BeyoECL Plus (Beyotime, China). Quantification of the detected bands was performed with Image-Pro Plus 6.0 software (Media Cybernetics Inc., USA). *β*-Actin was used as an internal control for total protein, and COX IV was used as an internal control for mitochondrial proteins.

### 2.4. Immunohistochemical Analysis

The auditory cortices from each group (*n* = 4) were collected. For immunohistochemical analysis and terminal deoxynucleotidyl transferase-mediated deoxyuridine triphosphate nick end labeling (TUNEL) staining, one hemisphere of the auditory cortex was fixed using 4% buffered paraformaldehyde (4% PFA) for 4 h at 4°C. For transmission electron microscopy (TEM) analysis, the other hemisphere of the auditory cortex was fixed using 2.5% glutaraldehyde for 4 h at 4°C. Following fixation in 4% PFA, the auditory cortex tissue was cryoprotected in 100 mM sodium phosphate buffer containing 40% sucrose at 4°C overnight. The auditory cortex was embedded in optimal cutting temperature (OCT) compound (Leica Microsystems, Germany) and sectioned on a cryostat at a thickness of 15 *μ*m. Sections were collected on 3-aminopropyl-trimethoxysilane-coated slides (Sigma-Aldrich, USA) and dried for 2 h in preparation for staining. Slides were washed in phosphate-buffered saline (PBS), incubated with 0.3% Triton X-100/PBS for 30 min, washed, blocked with 2% bovine serum albumin (BSA)/PBS, and washed again before incubation with anti-8-OHdG (1 : 200; mouse, Abcam, USA) and anti-COX IV (1 : 100; rabbit, Proteintech, China) antibodies overnight at 4°C. The slides were washed and incubated with anti-mouse Alexafluor-568 (1 : 500; goat, Invitrogen, USA) and anti-rabbit Alexafluor-488 (1 : 500; goat, Invitrogen, USA) secondary antibodies for 1 h at room temperature. After a final wash, sections were mounted with ProLong Gold antifade reagent with DAPI. Images were captured using a laser scanning confocal microscope (Leica TCS SP8, Germany). The expression of 8-OHdG was analyzed using Image-Pro Plus 6.0 software (Media Cybernetics Inc., Silver Spring, USA). The negative control sections were treated in the same manner, omitting the primary antibody incubation.

### 2.5. TEM

TEM was used to observe the ultrastructural alteration of the auditory cortex induced by T2DM. After postfixation in 1% osmium tetroxide for 2 h at room temperature, the auditory cortex tissue was dehydrated in an ascending graded series of ethanol and acetone, immersed in an acetone/Epon 812 mixture for 2 h, and immersed in Epon 812 for 2 h before being embedded in Epon 812 for 10 h at 80°C. Serial ultrathin sections (50 nm thick) were mounted on copper grids (200 mesh) and stained with uranyl acetate followed by lead citrate. The ultrastructure of the stained sections was examined under a transmission electron microscope (JEM-2100, JEOL Ltd., Japan).

### 2.6. Mitochondrial T-AOC Determination

Four rats from each group were sacrificed, and both hemispheres of the auditory cortex from each rat were rapidly removed. For mitochondrial T-AOC, ATP, and MMP detection, samples of the auditory cortex were homogenized in cold saline. Mitochondria in the auditory cortex were quickly extracted using a Tissue Mitochondria Isolation Kit (Beyotime, China) and subsequently used for the T-AOC. Mitochondrial T-AOC in the auditory cortex was determined using a Total Antioxidant Capacity Assay Kit (Beyotime, China) in combination with the fluorescence recovery after photobleaching method according to the manufacturer's instructions.

### 2.7. Detection of ATP Levels

The homogenized auditory cortex tissue was centrifuged at 4000 g for 15 min at 4°C, and the supernatant was used for ATP detection. Protein concentrations were detected using an Enhanced BCA Protein Assay Kit (Beyotime, China). ATP levels in the auditory cortex were quantified using colorimetric kits (Jiancheng, China) according to the manufacturer's instructions.

### 2.8. Measurement of Mitochondrial Membrane Potential (MMP)

To obtain the MMP measurement, the mitochondria of the auditory cortex were quickly extracted using a Tissue Mitochondria Isolation Kit (Beyotime, China). MMP levels in the auditory cortex were quantified using the fluorescent, lipophilic, and cationic probe, JC-1 (Jiancheng, China), according to the manufacturer's instructions.

### 2.9. TUNEL Staining

Apoptotic cells in the auditory cortex were detected in situ using a TUNEL POD kit (Roche Molecular Biochemicals, Germany). Slides were washed and incubated with 0.1% Triton X-100/sodium citrate for 2 min on ice. Slides were washed with PBS and labelled with a solution containing terminal deoxynucleotidyl transferase, its buffer, and fluorescein deoxyuridine triphosphate at 37°C for 1 h in a humidity chamber. Each slide was covered with ProLong Gold antifade reagent with DAPI and examined under a laser scanning confocal microscope (Leica TCS SP8, Germany). The number of TUNEL-positive stained cells were counted using Image-Pro Plus 6.0 software (Media Cybernetics Inc., USA).

### 2.10. Statistical Analysis

Data are expressed as mean ± standard deviation (SD), and the analysis was performed using SPSS 13.0 software (SPSS Inc., USA). Differences between the means of each group were analyzed using independent-samples *t*-test. The significance level was set at *P* < 0.05.

## 3. Results

### 3.1. ABR Thresholds Increased in ZDF Rats

**Figure 1:**
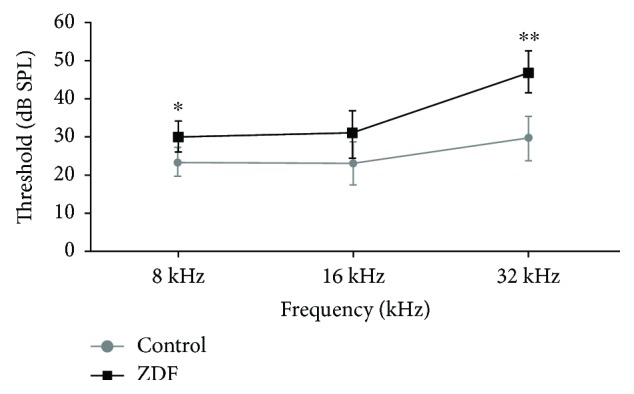
ABR thresholds of rats in the ZDF and control groups at 36 weeks. Data are expressed as the mean ± SD (*n* = 6). ^∗^*P* < 0.05 and ^∗∗^*P* < 0.01.

The auditory function of ZDF rats was detected by ABR to evaluate hearing impairment. The ABR thresholds of control rats at 8 kHz (low frequency), 16 kHz (middle frequency), and 32 kHz (high frequency) were 23.33 ± 3.76 dB SPL, 22.92 ± 5.79 dB SPL, and 29.58 ± 5.79 dB SPL, respectively. The ABR thresholds of ZDF rats at 8 kHz, 16 kHz, and 32 kHz were 30.00 ± 4.18 dB SPL, 30.42 ± 6.21 dB SPL, and 46.67 ± 5.16 dB SPL, respectively. The ABR thresholds of ZDF rats were significantly higher at 8 kHz and 32 kHz than those of control rats (Figure 1), which indicated that T2DM might induce auditory impairment at both low and high frequencies.

### 3.2. Protein Expression of NOX2 and Its Matching Subunits P22^phox^, P47^phox^, and P67^phox^ Increased in the Auditory Cortex of ZDF Rats

**Figure 2:**
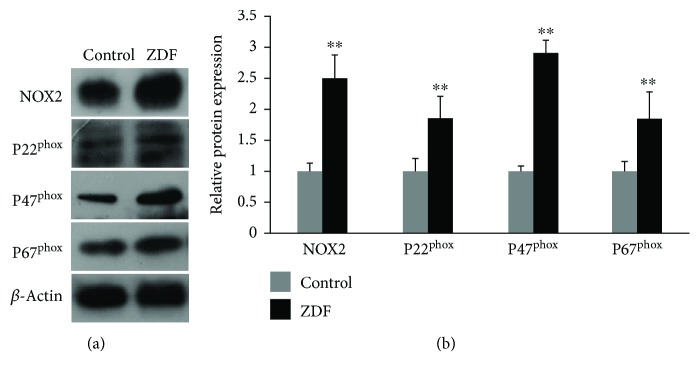
Protein expression of NOX2, P22^phox^, P47^phox^, and P67^phox^ in the auditory cortex of rats in the ZDF and control groups. (a) Representative images showing protein expression of NOX2, P22^phox^, P47^phox^, and P67^phox^ in the auditory cortex of rats in the ZDF and control groups using Western blot. (b) Quantification of NOX2, P22^phox^, P47^phox^, and P67^phox^ protein expression in the auditory cortex of rats in the ZDF and control groups. Data are expressed as mean ± SD (*n* = 4). ^∗∗^*P* < 0.01.

To evaluate NOX2-associated oxidative stress in the auditory cortex of ZDF rats, Western blot was used to detect NOX2 and its matching subunits P22^phox^, P47^phox^, and P67^phox^. Compared with the control group, protein expression of NOX2, P22^phox^, P47^phox^, and P67^phox^ in the ZDF group was increased by 2.51 ± 0.35-fold, 1.86 ± 0.35-fold, 2.92 ± 0.18-fold, and 1.86 ± 0.41-fold, respectively (*P* < 0.01) (Figures 2(a) and 2(b)), indicating that NOX2 might be a source of ROS generation in the auditory cortex of ZDF rats.

### 3.3. Mitochondrial DNA Oxidative Damage in the Auditory Cortex of ZDF Rats

**Figure 3:**
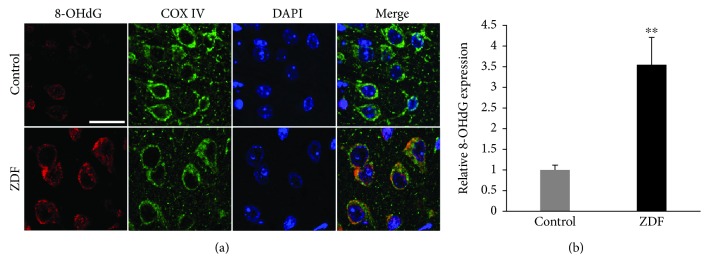
The expression of 8-OHdG in the auditory cortex of rats in the ZDF and control groups. (a) Representative images show the expression and localization of 8-OHdG (red) in the auditory cortex of rats in the ZDF and control groups using immunohistochemical staining. Scale bar = 20 *μ*m. (b) Quantification of 8-OHdG expression in the auditory cortex of rats in the ZDF and control groups. Data are expressed as mean ± SD (*n* = 4). ^∗∗^*P* < 0.01.

COX IV and 8-OHdG were detected by immunohistochemical analysis to evaluate mitochondrial DNA oxidative damage in the auditory cortex of ZDF rats. The slides of the auditory cortex were treated with 8-OHdG antibody to visualize DNA oxidative damage and COX IV antibody to visualize mitochondria. The expression of 8-OHdG (red) occurred mainly in the mitochondria (green) of neurons of the auditory cortex (Figure 3(a)). Compared with the control group, the quantitative analysis of 8-OHdG expression in the ZDF group was increased by 3.52 ± 0.66-fold (*P* < 0.01) (Figure 3(b)), suggesting that T2DM might induce oxidative damage of mitochondrial DNA in the auditory cortex.

### 3.4. Mitochondrial Dysfunction in the Auditory Cortex of ZDF Rats

**Figure 4:**
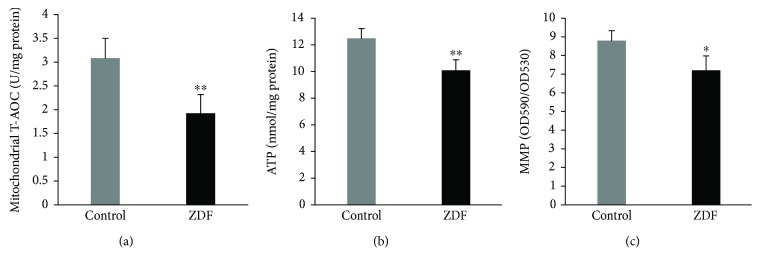
Levels of mitochondrial T-AOC, ATP, and MMP in the auditory cortex of rats in the ZDF and control groups. (a) Levels of mitochondrial T-AOC in the auditory cortex of rats in the ZDF and control groups. (b) Levels of ATP in the auditory cortex of rats in the ZDF and control groups. (c) Levels of MMP in the auditory cortex of rats in the ZDF and control groups. Data are expressed as mean ± SD (*n* = 4). ^∗^*P* < 0.05 and ^∗∗^*P* < 0.01.

The levels of mitochondrial T-AOC, ATP, and MMP were measured to evaluate mitochondrial function in the auditory cortex. The levels of mitochondrial T-AOC in the auditory cortex of the control group and ZDF group were 3.06 ± 0.41 U/mg protein and 1.92 ± 0.38 U/mg protein, respectively; the levels of mitochondrial T-AOC in the ZDF group were significantly lower than those in the control group (*P* < 0.01) (Figure 4(a)). The levels of ATP in the auditory cortex of the control group and ZDF group were 12.49 ± 0.59 nmol/mg protein and 9.98 ± 0.85 nmol/mg protein, respectively; the levels of ATP in the ZDF group were significantly lower than those in the control group (*P* < 0.01) (Figure 4(b)). The levels of MMP in the auditory cortex of the control group and ZDF group were 8.81 ± 0.49 and 7.19 ± 0.78, respectively; the levels of MMP in the ZDF group were also significantly lower than those in the control group (*P* < 0.05) (Figure 4(c)). These findings indicate that T2DM might induce mitochondrial dysfunction in the auditory cortex.

### 3.5. Ultrastructural Alteration in the Auditory Cortex of ZDF Rats

**Figure 5:**
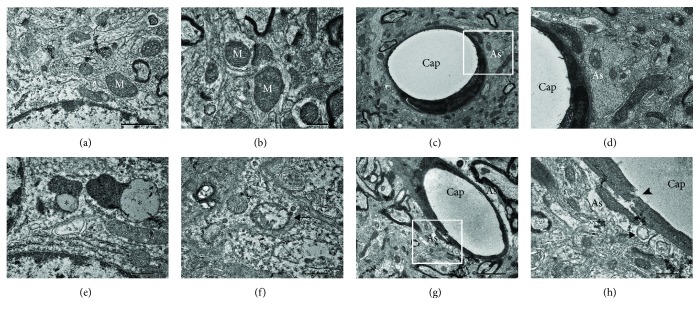
Ultrastructure in the auditory cortex of rats in the ZDF and control groups. (a, b) Normal mitochondria (white M) in the control group. (c, d) Normal profile of the neurovascular unit composed of astrocyte processes (white As) in the control group. (e) Lipofuscin granules (∗) in the ZDF group. (f) Enlarged mitochondria with reduced electron density in the matrix (arrow) in the ZDF group. (g, h) Neurovascular breakdown in the ZDF group: the disruption of capillary epithelia (arrowhead), enlarged astrocyte endfeet (black As), and severe degeneration of mitochondria (arrow). Scale bars: (a) = (b) = (c) = (e) = (f) = (h) = 0.5 *μ*m and (c) = (g) = 2 *μ*m. As: astrocyte processes; Cap: capillary.

The ultrastructure of the auditory cortex of rats was observed by TEM to evaluate the ultrastructural alteration in the auditory cortex of ZDF rats. In the control group, the shape and size of mitochondria were normal (Figures 5(a) and 5(b)) and the normal profile of neurovascular units was composed of astrocyte processes (Figures 5(c) and 5(d)). In the ZDF group, enlarged mitochondria with reduced electron density in the matrix (Figure 5(f)) and lipofuscin (Figure 5(e)) were frequently observed in the cytoplasm of neurons of the auditory cortex. Furthermore, perivascular astrocyte endfeet surrounding capillaries were enlarged and capillary epithelia were disrupted in the auditory cortex of ZDF rats (Figures 5(g) and 5(h)) and severe degeneration of mitochondria in enlarged perivascular astrocyte processes was also observed (Figure 5(h)). These findings indicated that T2DM might cause damage to mitochondria and neurovascular units in the auditory cortex.

### 3.6. Mitochondria-Dependent Apoptosis Activated in the Auditory Cortex of ZDF Rats

**Figure 6:**
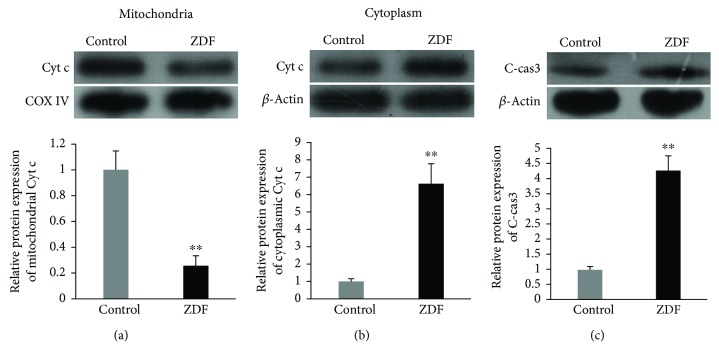
Mitochondria-dependent apoptosis in the auditory cortex of rats in the ZDF and control groups. (a) Protein expression of mitochondrial Cyt c in the auditory cortex of rats in the ZDF and control groups. (b) Protein expression of cytoplasmic Cyt c in the auditory cortex of rats in the ZDF and control groups. (c) Protein expression of C-cas3 in the auditory cortex of rats in the ZDF and control groups. Data are expressed as mean ± SD (*n* = 4). ^∗∗^*P* < 0.01.

**Figure 7:**
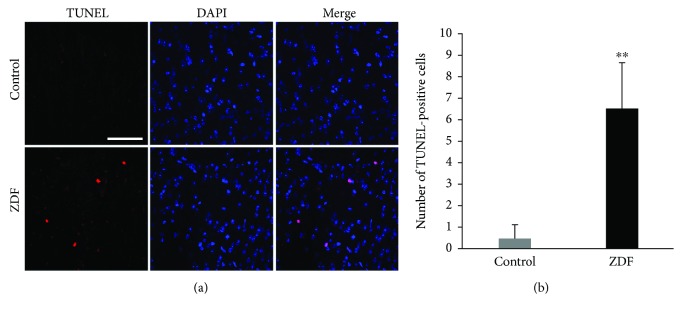
The occurrence of apoptosis in the auditory cortex of rats in the ZDF and control groups. (a) Representative images show the TUNEL-positive cells (red) in the auditory cortex of rats in the ZDF and control groups using TUNEL staining. Scale bar = 50 *μ*m. (b) Quantification of TUNEL-positive cells in the auditory cortex of rats in the ZDF and control groups. Data are expressed as mean ± SD (*n* = 4). ^∗∗^*P* < 0.01.

Mitochondrial and cytoplasmic Cyt c and C-cas3 were detected by Western blot to evaluate mitochondria-dependent apoptosis in the auditory cortex of ZDF rats. The mitochondrial protein levels of Cyt c in the ZDF group were 0.25 ± 0.08-fold compared with those in the control group (*P* < 0.01) (Figure 6(a)). The cytoplasmic protein levels of Cyt c in the ZDF group were 6.66 ± 1.12-fold compared with those in the control group (*P* < 0.01) (Figure 6(b)). The protein levels of C-cas3 in the ZDF group were 4.27 ± 0.49-fold compared with those in the control group (*P* < 0.01) (Figure 6(c)). To further evaluate the occurrence of apoptosis in the auditory cortex of ZDF rats, the apoptotic cells were detected by TUNEL staining (Figure 7(a)). The numbers of TUNEL-positive cells in the control group and ZDF group were 0.50 ± 0.58 and 6.50 ± 2.08, respectively. The number of TUNEL-positive cells in the ZDF group was significantly higher than that in the control group (*P* < 0.01) (Figure 7(b)). These results indicated that the mitochondria-dependent apoptosis was activated in the auditory cortex of ZDF rats.

## 4. Discussion

T2DM is known to cause microvascular damage and neuropathy, primarily affecting the peripheral arteries and nerves [22]. The cochlear and auditory nerves are similarly at risk. Previously, it has been found that, compared to controls, diabetic patients demonstrated a more significant reduction in cochlear hair cells and ticker vessel walls, in addition to causing greater atrophy of the stria vascularis [6]. The recent study demonstrated that auditory impairment in ZDF rats, a T2DM rodent model, was accompanied by ultrastructural damage to the stria vascularis of the cochlea [20].

Additionally, a meta-analysis involving eighteen studies found that diabetic patients had a 3-fold delay in the ABR latency of wave V, suggesting a reduction in the function of inferior colliculi in the CAS [3]. The present results demonstrate that mitochondrial ultrastructural damage and neurovascular unit breakdown in the auditory cortex of the CAS might also be partly responsible for T2DM-induced hearing loss. The cochlea is the most peripheral part along the auditory pathway, whereas the auditory cortex is the apex auditory center for coding and processing acoustic information. Changes in the CAS may affect the ability to localize temporal and spatial origins of sounds and impair speech comprehension in noisy environments [23].

Oxidative stress brought on by hyperglycemia has been shown to result in neural damage [24–26]. Previously, we have demonstrated that overexpression of NOX was a main source of ROS in the cochlea of rats fed with a high-fat diet for 12 months; we also observed mitochondrial ultrastructural damage in the stria vascularis of the cochlea of rats fed with the high-fat diet [9]. In this study, we report, for the first time, that the levels of NOX2 and its matching subunits P22^phox^, P47^phox^, and P67^phox^ were significantly increased in the auditory cortex of ZDF rats. As opposed to mitochondria, which generate ROS as a byproduct of their metabolism, the NOX system is a direct ROS generator [27]. Previous reports show that NOX2-associated oxidative stress may be responsible for ultrastructural damage leading to the dysfunction of the central nervous system due to aging, ischemia-reperfusion, and drugs [28–30]. Furthermore, previous studies demonstrated that activation of NOX2 may induce oxidative stress and cell dysfunction in *in vitro* and *in vivo* models of glucolipotoxicity and diabetes [31] and that T2DM was strongly associated with increased vascular NOX-generated ROS in humans [32]. Therefore, overexpression of NOX2 and its matching subunits in the auditory cortex of ZDF rats may indicate that NOX2-generated ROS may play an important role in the process of T2DM-induced CAS impairment.

Mitochondria are particularly susceptible to ROS-induced oxidative damage because of the lack of protective histones [33]. Oxidative damage to mitochondrial DNA is much greater compared to the damage to nuclear DNA [34]. The present study indicated that 8-OHdG, a marker of DNA oxidative damage, was mainly located in the neuronal mitochondria of the auditory cortex of ZDF rats, indicating that T2DM might induce oxidative damage to mitochondrial DNA in the CAS. To further investigate mitochondrial oxidative damage in the auditory cortex of ZDF rats, the mitochondrial ultrastructure was analyzed using TEM and degenerated mitochondria in the neurons and enlarged perivascular astrocyte processes were observed. Oxidative damage to mitochondrial DNA and mitochondrial ultrastructure in the auditory cortex may lead to mitochondrial dysfunction, leading to the decline of mitochondrial T-AOC, ATP production, and MMP levels.

Moreover, mitochondrial dysfunction in neurons may activate the mitochondria-dependent apoptotic pathway in the CAS [35, 36]. Besides damaging mitochondria, oxidative stress also impairs the normal function of astrocytes [37]. Astrocyte endfeet, as components of the neurovascular unit, play an essential role in maintaining homeostasis of the brain microenvironment [38]. Therefore, enlarged perivascular astrocyte endfeet surrounding capillaries and the disruption of capillary epithelia in the auditory cortex of ZDF rats suggested that T2DM may induce microenvironment impairment of CAS.

**Figure 8:**
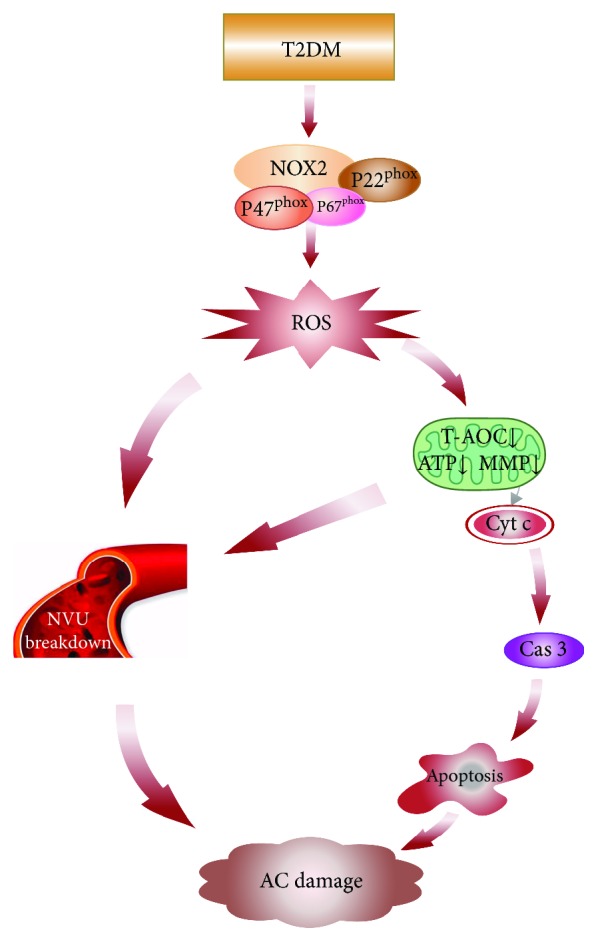
Schematic diagram of auditory cortex damage induced by T2DM. T2DM induces NOX2 and its corresponding subunits form an active NOX2 complex to promote ROS generation. ROS overproduction induces NVU breakdown and mitochondrial oxidative damage including T-AOC decline and mitochondrial dysfunction (ATP↓ and MMP↓). The impaired mitochondria release Cyt c from the mitochondrial intermembrane space into the cytoplasm and induced apoptosis via the caspase 3-dependent pathway. Meanwhile, the mitochondrial dysfunction also triggers NUV breakdown. Finally, the neuronal apoptosis and NUV breakdown may lead to AC damage. AC: auditory cortex; Cas 3: caspase 3; Cyt c: cytochrome c; MMP: mitochondrial membrane potential; NOX2: NADPH oxidase 2; NVU: neurovascular unit; ROS: reactive oxygen species; T2DM: type 2 diabetes mellitus; T-AOC: total antioxidant capabilities.

In summary, the present data provide novel evidence that NOX2-associated mitochondrial oxidative damage and neurovascular unit breakdown in the auditory cortex of CAS may be partly responsible for T2DM-induced auditory impairment (Figure 8). Therefore, NOX2 may be a useful therapeutic target in treating hearing loss associated with T2DM and neurodegenerative diseases.

## Data Availability

The data used to support the findings of this study are available from the corresponding author upon request.

## Abbreviations

8-OHdG: 8-Hydroxy-2-deoxyguanosine
ABR: Auditory brainstem response
ATP: Adenosine triphosphate
CAS: Central auditory system
C-cas3: Cleaved caspase 3
COX: Cytochrome c oxidase
Cyt c: Cytochrome c
NOX: NADPH oxidase
MMP: Mitochondrial membrane potential
ROS: Reactive oxygen species
T2DM: Type 2 diabetes mellitus
T-AOC: Total antioxidant capabilities
TEM: Transmission electron microscopy
TUNEL: Terminal deoxynucleotidyl transferase-mediated deoxyuridine triphosphate nick end labeling
ZDF: Zucker diabetic fatty.
